# Association of dietary patterns with depressive symptoms in Chinese adolescents: a cross-sectional study

**DOI:** 10.3389/fnut.2023.1180858

**Published:** 2023-07-11

**Authors:** Kongjia Qian, Dan Wang, Yongfang Sun, Xian Ye, Dan Wang, Hongzhen Xu

**Affiliations:** ^1^Nursing Department, Children’s Hospital, Zhejiang University School of Medicine, National Clinical Research Center for Child Health, Hangzhou, China; ^2^Department of Paediatrics, Taizhou Hospital of Zhejiang Province Affiliated to Wenzhou Medical University, Linhai, China

**Keywords:** depression, dietary patterns, adolescents, China, cross-sectional study

## Abstract

**Objective:**

Depression is highly prevalent in adolescents and may have adverse social and health consequences. To investigate the effect of diet on the occurrence of depression in adolescents, this study examined the correlation between dietary patterns and depression in adolescents.

**Methods:**

A total of 853 participants were recruited in September to October 2022 in Taizhou, Zhejiang, China. The Chinese version of the Patient Health Questionnaire-9 was used to assess the subjects’ depressive symptoms in the past 2 weeks. Relevant dietary information was obtained through a food frequency questionnaire. The associations between dietary patterns and the risk of depression were assessed using a logistic regression model.

**Results:**

Four dietary patterns were identified by factor analysis, including the modern pattern, the snack-aquatic pattern, the traditional pattern, and the vegetarian pattern. The risk of mild depression among adolescents was higher in the Q2 and Q3 groups of the modern model than in the Q1 group of this type, and the risk of mild depression was higher in the Q4 group of the snack-aquatic model than in the Q1 group of this type; while the risk of moderate depressive symptoms was lower in the Q3 group of the vegetarian model compared with the Q1 group.

**Conclusion:**

Modern and snack-aquatic patterns are associated with an increased risk of depression in Chinese adolescents, and vegetarian patterns are associated with a reduced risk of depression. The current findings emphasize the importance of adolescents consuming moderate amounts of rice flour, adequate vegetables and fruits, and reducing snack intake.

## Introduction

Depression is one of the most common mental illnesses in the world and is a growing social and health problem today (1). It is also an important influence on problematic behaviors such as suicide, smoking, alcohol abuse, reduced learning ability and aggression in adolescents (2). It is estimated that 322 million people worldwide suffer from depression (3), and 34% of adolescents are at risk of clinical depression (4) and is one of the leading causes of the global burden of disease in adolescents (5). The prevalence of major depression among adolescents in the United States is 3%, with a lifetime prevalence of around 11% (6). Chinese data show that the overall detection rate of depressive symptoms among secondary school students is 28.4% (2) and the overall trend of depression levels is increasing (7). However, the public recognition rate of depression in China is only 25.4% (8), only 0.5% of depressed patients receive adequate treatment (9). Therefore it is urgent and important to increase attention to adolescent depression, identify and avoid risk factors, diagnose early and treat effectively.

Dietary characteristics as an influencing factor in depressive symptoms (10), has attracted much attention in recent years. However, existing studies have produced mixed results and lack definitive conclusions. A meta-analysis found that after summarizing 21 articles on the relationship between dietary patterns and depression dietary patterns characterized by a high intake of fruit, vegetables, whole grains, fish, olive oil, low-fat dairy products and antioxidants were associated with a reduced risk of depression (11). A Korean study showed that consumption of fast food, including lamian noodle, burgers, pizza, fried foods and other processed foods, was associated with an increased risk of depression in adolescent girls (12). However, some studies have also observed inconsistent results in that depressive symptom severity was not independently associated with dietary patterns (13). For this reason, we explored the correlation between different dietary patterns and depression in adolescents.

## Methods

### Design and study population

This study is a cross-sectional survey study. From September 29 to October 5, 2022, in an urban area of Taizhou, Zhejiang, China, a randomly selected middle school, using stratified whole group sampling, all seventh and eighth grade students were selected as the study population. A total of 1,061 questionnaires were distributed in this study, 881 questionnaires were returned, and after excluding invalid questionnaires, a total of 853 valid questionnaires were finally included, the effective rate is 80.40%. All subjects and guardians provided written informed consent and the study protocol was approved by the hospital ethics committee (No. K20220906).

### Questionnaires

The students had no mobile phone, so the research group made the two-dimensional code of the questionnaire into a card. The students who were surveyed would bring the card back after physical measurement, and use the guardian’s mobile phone to fill in the electronic questionnaire anonymously. The survey included general information (gender, grade, age, family residence, type of family, residence status, whether the child was the only child, parental education, number of sports, number of sleeping hours, height, and body mass), diet and depressive symptoms.

### Physical measurements

In this study, height and body mass were included, and body mass index (BMI) was calculated. Complete the above measurements in school. A uniformly calibrated weight scale and height ruler were used and the subjects were asked to stand barefoot with only a small amount of clothing on, heels together about 45 degrees apart, arms down, body naturally straight, eyes forward, shoulders, hips and heels close to the ruler, and height and body mass were measured and read by four nurses who were uniformly trained to read height and body mass. After the physical measurement was completed, the data were checked by two nurses and filled out on the card of the questionnaire, which was brought back by the students. Students were asked to fill in these data in the questionnaire.

### Assessment of dietary patters

Diet was assessed by a comprehensive food frequency questionnaire (FFQ), was adapted from the Chinese version of FFQ for adolescents (14), which designed to measure the dietary habits of adolescents over the last 3 months. Single foods in the FFQ were divided into 13 pre-defined food groups based on similarity in nutritional composition and cooking methods (Table 1). Students were asked to choose whether to eat each food, how often to eat it, how many times to eat it and how many servings to eat. There were four options for frequency of eating: 7 = eat every day, 1 = eat every week, 0.25 = eat every month and 0 = eat every year. The choice of number of servings was determined by the frequency of eating, with students choosing the number of servings per day/week/month/year. For this study, the same food container was used as a reference, the amount of each serving of each food group was standardized and made into a picture, and students were asked to choose the average number of servings they ate per serving based on the picture. Frequency of eating × number of times eaten × number of servings/7 = total number of servings per day, i.e., the average total number of servings per day for each food group was obtained.

**Table 1:** Food grouping used in dietary pattern analysis.

Food Groups	Food items
Rice flour	Rice, buns, noodles, pancakes, bread
Livestock meat	Chicken and duck, pork, beef and lamb
Eggs	Eggs, duck eggs
Legumes	Soybean milk, soybean skin, tofu, dried beans
Aquatic products	Fish, shrimp, crab, squid
Dairy products	Plain milk, yogurt, cheese
Non-starchy vegetables	Spinach, Chinese cabbage, mini Chinese cabbage, beans, tomatoes, carrots
Root vegetables	Potatoes, lotus root
Bacteria and algae	Needle mushrooms, mushrooms and seaweed
Nuts	Melon seeds, Badam, almonds, cashews
Rough food	Oats, maize, sweet potato
Fruit	Apples, oranges, pears, blueberries, dragon fruit
Snacks	Instant noodles, sweets, fried snacks

### Assessment of depressive symptoms

Depressive symptoms were measured using the Chinese version of the patient health questionnaire-9 (PHQ-9), which has good reliability and validity when applied to the assessment of depressive mood in Chinese adolescents (15). The Cronbach’s alpha coefficient is 0.85, retest reliability is 0.88. It assesses feelings over the past 2 weeks, There were 9 entries each entry rated on a four-point scale from 0 (not at all) to 3 (almost every day). The total questionnaire score was the sum of the 9 entries’ scores, scores ranging from 0 to 27, higher scores indicating more severe depressive symptoms.

### Statistical analysis

EXCEL was applied for data processing and SPSS version 27.0 (IBM, Armonk, NY, United States) was used for statistical analysis. The characteristics of participants with and without depression were compared using the *t*-test for continuous variables and chi-squared test for categorical variables. Variables that were statistically different between the depressed and non-depressed groups in the univariate analysis (*p* < 0.05) were further included in the logistic regression model by the “stepwise forward method” and the odds ratio (OR) and 95% confidence interval (95% CI) were calculated.

Factor analysis was used to obtain the dietary patterns of the survey population. Kaiser–Meyer–Olkin (KMO) test and Bartlett’s test of sphericity were used to assess the suitability of the data for factor analysis. A more explanatory factor model is obtained by variance maximization orthogonal rotation. The number of factors was retained based on an evaluation of the eigenvalues (>1.0), a scree plot, and factor interpretability. The factor (dietary pattern) was named by the food category with factor loadings >0.3. The factor scores corresponding to each dietary pattern were calculated by regression. The quartile method was used to divide the factor scores of each dietary pattern into four equal parts, Q1: factor score <*P*25; Q2: factor score intermediate *P*25–*P*50; Q3: factor score intermediate *P*50–*P*75; Q4: factor score >*P*75.

The association between dietary pattern and depressive symptoms was obtained using depressive symptoms (no depression: 0, mild depression: 1, moderate and above depression: 2) as the dependent variable and the dietary pattern quartile grouping as the independent variable in a logistic regression model, after adjusting for selected covariates. The test level for this study was *α* = 0.05.

## Results

### Sociodemographic characteristics of the survey respondents

A total of 853 individuals were included in this study, 474 (55.6%) males and 379 (44.4%) females. The mean age of the participants was 13.10 ± 0.70 years. The overall prevalence of depression in this population was 29.07%. Comparing the sociodemographic characteristics of the depressed and non-depressed groups, the results are shown in Table 2, with statistical differences between the two groups in terms of grade, gender, age and family type (*p* < 0.05).

**Table 2:** Description of basic information about the students in the depressed and non-depressed groups.

Characteristics		Total	Depression		*p*
			No	Yes	
Grade level	Grade 7	457 (53.6)	363 (60.0)	94 (37.9)	<0.001
	Grade 8	396 (46.4)	242 (40.0)	154 (62.1)	
Gender	Male	474 (55.6)	353 (58.3)	121 (48.8)	0.011
	Female	379 (44.4)	252 (41.7)	127 (51.2)	
Age (y)		13.10 ± 0.70	13.04 ± 0.60	13.23 ± 0.88	0.003
Height (cm)		157.63 ± 7.64	157.43 ± 7.77	158.10 ± 7.31	0.245
Body weight (kg)		50.43 ± 11.91	50.54 ± 12.29	50.17 ± 10.94	0.686
BMI (kg/m^2^)		20.17 ± 3.85	20.25 ± 3.99	19.96 ± 3.47	0.317
Residential	No	632 (74.1)	453 (74.9)	179 (72.2)	0.414
	Yes	221 (25.9)	152 (25.1)	69 (27.8)	
The only child	No	718 (84.2)	515 (85.1)	203 (81.9)	0.235
	Yes	135 (15.8)	90 (14.9)	45 (18.1)	
Place of residence	Rural	392 (46.0)	270 (44.6)	122 (49.2)	0.259
	Township	449 (52.6)	328 (54.2)	121 (48.8)	
	City	12 (1.4)	7 (1.2)	5 (2.0)	
Type of family	Two-parent families	697 (81.7)	520 (86.0)	177 (71.4)	<0.001
	Single parent families	120 (14.1)	68 (11.2)	52 (21.0)	
	Reconstituting the family	36 (4.2)	17 (2.8)	19 (7.7)	
Living condition	Living with parents	464 (54.4)	327 (54.0)	137 (55.2)	0.183
	Living with grandparents	243 (28.5)	179 (29.6)	64 (25.8)	
	Living with parents and grandparents	123 (14.4)	87 (14.4)	36 (14.5)	
	Living with relatives	23 (2.7)	12 (2.0)	11 (4.4)	
Father’s qualifications	Primary school and below	135 (15.8)	88 (14.5)	47 (19.0)	0.603
	Lower secondary	532 (62.4)	382 (63.1)	150 (60.5)	
	High school/secondary School	163 (19.1)	119 (19.7)	44 (17.7)	
	Tertiary	16 (1.9)	11 (1.8)	5 (2.0)	
	Undergraduate	7 (0.8)	5 (0.8)	2 (0.8)	
	Master and above	0			
Mother’s qualifications	Primary school and below	180 (21.1)	123 (20.3)	57 (23.0)	0.059
	Lower secondary	475 (55.7)	330 (54.5)	145 (58.5)	
	High School	169 (19.8)	135 (22.3)	34 (13.7)	
	Tertiary	23 (2.7)	13 (2.1)	10 (4.0)	
	Undergraduate	5 (0.6)	3 (0.5)	2 (0.8)	
	Masters and above	1 (0.1)	1 (0.2)	0	
Monthly family income (CNY)	<5,000	406 (47.6)	277 (45.8)	129 (52.0)	0.535
	5,000–9,999	335 (39.3)	244 (40.3)	91 (36.7)	
	10,000–14,999	88 (10.3)	66 (10.9)	22 (8.9)	
	15,000–19,999	14 (1.6)	11 (1.8)	3 (1.2)	
	≥20,000	10 (1.2)	7 (1.2)	3 (1.2)	
Number of exercises	<3	157 (18.4)	113 (18.7)	44 (17.7)	0.749
	≥3	696 (81.6)	492 (81.3)	204 (82.3)	
Exercise time	<30 min	342 (40.1)	240 (39.7)	102 (41.1)	0.697
	30–60 min	446 (52.3)	316 (52.2)	130 (52.4)	
	>60 min	65 (7.6)	49 (8.1)	16 (6.5)	
Sleep time (h)		8.55 ± 2.28	8.64 ± 2.19	8.33 ± 2.46	0.064

Mean ± SD or *n* (%), CNY, Chinese Yuan.

### Factors influencing depressive symptoms among survey respondents

A binary logistic stepwise regression analysis was conducted using whether or not depression was the dependent variable and the four variables mentioned above that differed between the depressed and non-depressed groups in terms of grade, gender, age and family type as independent variables. The results showed that depressive symptoms were 2.457 times more likely to occur in grade 8 than in grade 7; girls were more likely to be depressed, 1.47 times than boys; single-parent families were 2.247 times than two-parent families, and reconstituted families were 3.283 times than two-parent families; and the probability of depression increased by 0.574 times for each additional year of age within this age range (Table 3).

**Table 3:** Factors influencing depressive symptoms.

Variables	*β*	*S*_χ-_	Wald*χ*^2^	*p*	OR (95% CI)
Grade level					
Grade 7	0	0	–	–	1.000
Grade 8	0.899	0.155	33.658	<0.01	2.457 (1.814, 3.33)
Gender					
Male	0	0	–	–	1.000
Female	0.385	0.151	6.476	0.011	1.470 (1.093, 1.978)
Age (y)	0.453	0.125	13.213	<0.01	1.574 (1.232, 2.009)
Type of family					
Two-parent families	0	0	–	–	1.000
Single parent families	0.809	0.204	15.783	<0.01	2.247 (1.507, 3.349)
Reconstituting the family	1.189	0.345	11.875	<0.01	3.283 (1.67, 6.457)

### Analysis of dietary patterns

The factor-loading matrices for each dietary pattern are shown in Table 4. The dietary pattern was established using factor analysis with KMO = 0.743, *p* < 0.001 in Bartlett’s sphericity test, which qualified for factor analysis. Four dietary patterns were identified. Pattern 1, which high intake of legumes, aquatic products, bacteria and algae, nuts, rough food and low intake of rice flour, was labelled the modern pattern. Pattern 2, with high loadings for aquatic products, dairy products, snacks and low loadings for rice flour, vegetables, was labelled the snack-aquatic pattern. Pattern 3, with high loadings for rice flour, livestock meat, eggs, non-starchy vegetables, was labelled the traditional pattern. Pattern 4, with high loadings for dairy products, non-starchy vegetables, fruit, was labelled the vegetarian pattern. These four dietary patterns explained 50.81% of the variance of the food groups.

**Table 4:** Factor loadings for four dietary patterns derived from a principal component analysis.

Food groups	Modern pattern	Snack-aquatic pattern	Traditional pattern	Vegetarian pattern
Rice flour	−0.054	−0.174	**0.755**	0.028
Livestock meat	0.079	0.234	**0.657**	0.189
Eggs	0.224	0.095	**0.42**	−0.003
Legumes	**0.665**	0.217	0.248	−0.195
Aquatic products	**0.377**	**0.67**	0.144	−0.133
Dairy products	0.076	**0.411**	0.148	**0.529**
Non-starchy vegetables	0.201	−0.224	**0.311**	**0.556**
Root vegetables	**0.67**	0.001	0.028	0.215
Bacteria and algae	**0.635**	0.015	0.158	0.113
Nuts	**0.563**	−0.047	−0.212	0.298
Rough food	**0.663**	0.07	0.087	0.065
Fruit	0.106	0.079	−0.028	**0.676**
Snacks	−0.092	**0.832**	−0.038	0.157

The bold value indicates that the item is one of the representative foods under the corresponding dietary pattern.

### Relationship between dietary patterns and depressive symptoms

The relationship between the four dietary patterns and depressive symptoms is shown in Table 5. Model 2 adjusted for grade, age and gender, with the modern pattern group Q2 & group Q3 having a higher risk of mild depressive symptoms compared to group Q1, with the snack-aquatic pattern having a higher risk of mild depressive symptoms compared to group Q1, however group Q3 in the vegetarian pattern compared to group Q1 having a lower risk of moderate depressive symptoms. After further adjusting for variables such as whether or not they lived in school, whether or not they were the only child, place of residence, height (cm), weight (kg), BMI, family type, family education level, living condition, family structure, monthly family income, frequency of exercise per week, time spent per exercise session, and sleep duration (i.e., model 3), the Q2 and Q3 groups of the modern pattern compared to the Q1 group, and the Q4 group of the snack-aquatic pattern compared to the Q1 group, the association of a higher risk of mild depressive symptoms remained; no association was observed between the traditional and vegetarian models and the risk of depressive symptoms.

**Table 5:** Analysis of the relationship between dietary patterns and depressive symptoms.

Dietary patterns	Quartiles of dietary patterns	Sample size	OR (95% CI)					
			Model 1		Model 2		Model 3	
			Mild	Moderate and above	Mild	Moderate and above	Mild	Moderate and above
Modern pattern	Q1	214	1.000 (Ref)	1.000 (Ref)	1.000 (Ref)	1.000 (Ref)	1.000 (Ref)	1.000 (Ref)
	Q2	213	1.597 (0.975, 2.617)	0.632 (0.321, 1.246)	1.694 (1.022, 2.808)^*^	0.609 (0.303, 1.225)	1.799 (1.063, 3.047)^*^	0.620 (0.302, 1.272)
	Q3	212	1.651 (1.009, 2.703)^*^	0.641 (0.325, 1.264)	1.693 (1.024, 2.801)^*^	0.632 (0.318, 1.256)	1.861 (1.099, 3.151)^*^	0.637 (0.314, 1.292)
	Q4	214	1.220 (0.731, 2.035)	0.765 (0.402, 1.446)	1.284 (0.762, 2.164)	0.774 (0.406, 1.476)	1.382 (0.802, 2.382)	0.737 (0.380, 1.430)
Snack-aquatic pattern	Q1	213	1.000 (Ref)	1.000 (Ref)	1.000 (Ref)	1.000 (Ref)	1.000 (Ref)	1.000 (Ref)
	Q2	214	0.909 (0.546, 1.514)	1.091 (0.565, 2.109)	0.889 (0.529, 1.494)	1.040 (0.531, 2.037)	0.856 (0.501, 1.461)	1.050 (0.526, 2.094)
	Q3	213	1.173 (0.720, 1.910)	0.800 (0.392, 1.631)	1.130 (0.687, 1.857)	0.772 (0.376, 1.586)	1.166 (0.699, 1.946)	0.777 (0.374, 1.614)
	Q4	213	1.708 (1.067, 2.734)^*^	1.139 (0.579, 2.239)	1.700 (1.053, 2.747)^*^	1.123 (0.567, 2.224)	1.724 (1.043, 2.849)^*^	1.035 (0.511, 2.095)
Traditional pattern	Q1	214	1.000 (Ref)	1.000 (Ref)	1.000 (Ref)	1.000 (Ref)	1.000 (Ref)	1.000 (Ref)
	Q2	213	0.764 (0.477, 1.224)	0.514 (0.262, 1.010)	0.794 (0.490, 1.287)	0.536 (0.269, 1.065)	0.877 (0.531, 1.447)	0.618 (0.305, 1.253)
	Q3	213	0.550 (0.334, 0.906)^*^	0.518 (0.267, 1.005)	0.608 (0.365, 1.013)	0.577 (0.294, 1.133)	0.647 (0.380, 1.101)	0.702 (0.348, 1.415)
	Q4	213	0.998 (0.632, 1.574)	0.627 (0.326, 1.206)	1.134 (0.707, 1.820)	0.711 (0.363, 1.394)	1.242 (0.756, 2.038)	0.807 (0.399, 1.630)
Vegetarian pattern	Q1	214	1.000 (Ref)	1.000 (Ref)	1.000 (Ref)	1.000 (Ref)	1.000 (Ref)	1.000 (Ref)
	Q2	212	0.614 (0.379, 0.995)^*^	0.567 (0.285, 1.129)	0.616 (0.377, 1.008)	0.536 (0.265, 1.086)	0.654 (0.391, 1.092)	0.572 (0.276, 1.182)
	Q3	213	0.640 (0.397, 1.031)	0.448 (0.215, 0.933)^*^	0.617 (0.379, 1.003)	0.423 (0.202, 0.888)^*^	0.645 (0.387, 1.074)	0.475 (0.222, 1.017)
	Q4	214	0.954 (0.604, 1.506)	1.036 (0.559, 1.922)	0.915 (0.573, 1.460)	1.010 (0.540, 1.890)	0.913 (0.557, 1.497)	1.076 (0.561, 2.064)

Q, quartile; Q1 (<*P*25), Q2 (*P*25–*P*50), Q3 (*P*50–*P*75), Q4(>*P*75); OR, odds ratio; CI, confidence interval. Model 1: unadjusted; model 2: adjusted for grade, age and gender; model 3: adjusted for grade, age, gender, whether living in school, whether only child, place of residence, height (cm), weight (kg), BMI, family type, family education level, residence status, family structure, monthly family income, frequency of exercise per week, duration of exercise per session, and sleep duration. ^*^p < 0.05.

## Discussion

The detection rate of depression among adolescents in this study was 29.07%, while related studies in China showed that the detection rate of depressive symptoms among adolescents ranged from 6.4% to 60.7% (2). The differences in the detection rates of depression among adolescents in different studies may be related to the different socio-cultural and economic backgrounds of the studies and the different assessment tools used. The results of this study showed that grade, age, gender, and family structure were influential factors in the development of depressive symptoms, a finding that is similar to existing studies (10, 16).

Four main dietary patterns were identified using principal component analysis and factor analysis: modern pattern, snack-aquatic pattern, traditional pattern, and vegetarian pattern. The four dietary patterns explained 22.18%, 10.41%, 9.84%, and 8.38% of the total variance in food intake, respectively, explaining a total of 50.81% of the variance, which is higher than previous studies on dietary patterns of Chinese adolescents explained 35% (17) and 49.5% (18) of the total variance in food intake in the previous study. This indicates that the dietary patterns derived from this study based on principal component analysis are more consistent with the actual situation of adolescents.

The study found that adolescents who followed the modern pattern (high intake of legumes, aquatic products, root vegetables, bacteria and algae, nuts, rough food and low intake of rice flour) and the snack-aquatic pattern (high intake of aquatic products, dairy products, snacks and low intake of rice flour and vegetables) had a higher risk of mild depressive symptoms, while adolescents who followed the vegetarian pattern (high intake of fruit, non-starchy of vegetables and dairy products) had a lower risk of moderate and above depressive symptoms. In the snack-fish pattern, the factor loading for aquatic products was higher, but, previous studies have associated aquatic products consumption with a lower risk of depression (19), which may be related to the fact that Chinese cooking methods are different from those of other countries, as it is customary to fry, deep-fry and grill aquatic products in China. However, there is no information related to cooking methods in this study, so the specific effects of cooking factors need to be further explored. There is now a lot of evidence that the diet that has a positive effect on depression is the Mediterranean diet (MD), with vegetables and fruit being the main components of the MD (1); a case-control study in Korea showed that consumption of green vegetables could reduce the risk of depression in adolescent girls (12). These studies have some similarities to the vegetarian pattern in this article. The results of this study suggest that intake of processed foods, commercial baked goods and confectionery should be limited, as confirmed by studies in Korea, Australia and Spain (12, 20, 21). These foods are rich in trans fatty acids, saturated fats, refined carbohydrates and added sugars, and are low in nutrients and fibre (22). The results of this study emphasise the importance of adolescents consuming moderate amounts of rice flour, adequate vegetables and fruit, and reducing snack intake.

Indeed, the associations between dietary patterns and depressive symptoms are complex and multifaceted. In our study, we found that the relationships between quartile groups of dietary patterns and depressive symptoms were not strictly linear and there may be a threshold effect at play. This means that the relationship between dietary patterns and depressive symptoms may be more pronounced for individuals who consume a relatively high or low amount of a particular dietary pattern, rather than those who consume moderate amounts. For example, it is possible that the relationship between the modern dietary pattern and depressive symptoms may be stronger for individuals who consume a high amount of highly processed and refined foods, compared to those who consume moderate amounts. Moreover, the specific components and nutrient content of different dietary patterns can vary widely, and this can make it challenging to tease apart the specific factors that may be driving observed associations. For example, it is possible that the differences in the associations between quartile groups and depressive symptoms that we observed may be due to differences in nutrient intake, lifestyle factors, or other confounding variables that were not captured in our analysis.

This study has the following strengths: firstly, the study selected a population of adolescents at higher risk of depressive symptoms and greater health risks, a population for which there are currently few studies in China; secondly, this study included 7th and 8th grade students as respondents and did not include the 9th grade students, who were about to sit for the mid-term examinations, also the study was conducted during the National Day holiday, when the COVID-19 in China was made the agreements on implementing regular epidemic prevention and control measures, in order to excluding the effects of academic stress and the COVID-19 on depression as far as possible; thirdly, the dietary patterns derived from the principal components analysis matched the actual situation of adolescents to a high degree; fourthly, the study adjusted for sociodemographic variables, reducing the possibility of confounding. However, there are certain limitations. Firstly, the survey respondents were from only one secondary school and the findings have limited external validity. Secondly, when conducting the analysis of dietary patterns, it was only possible to analyse by broad food groups, which has limitations in identifying unhealthy foods.

Thirdly, the scores of each dietary pattern only represent the relative weights of that pattern and cannot fully reflect participants’ intake levels in other dietary patterns. The overlap among the different eating patterns may complicate the interpretation of our findings and make it more difficult to isolate the specific effects of each dietary pattern. Finally, although many possible covariates were considered in this study, it cannot be excluded that all unknown factors had an impact on the associations observed so far.

## Conclusion

Adolescent depression is a mental illness that urgently needs our attention and intervention. This study on the relationship between dietary patterns and depressive symptoms suggests that vegetarian patterns are associated with a reduced risk of depression, and modern and snack-aquatic patterns are associated with an increased risk of depression. Future research is needed to further tease apart the specific factors that may be driving observed associations, as well as the possibility of threshold effects, and to identify potential strategies for preventing and treating depression through dietary interventions.

## Data availability statement

The raw data supporting the conclusions of this article will be made available by the authors, without undue reservation.

## Ethics statement

The studies involving human participants were reviewed and approved by Taizhou Hospital of Zhejiang Province, China (No. K20220932). Written informed consent to participate in this study was provided by the participants’ legal guardian/next of kin.

## Author contributions

KQ data analysis, and draft the initial manuscript. DW and YS data collection, DW provide data. XY data statistics. DW research design, data collection supervision, validation part of the statistical work. HX resource and supervision. All authors contributed to the article and approved the submitted version.

## Conflict of interest

The authors declare that the research was conducted in the absence of any commercial or financial relationships that could be construed as a potential conflict of interest.
